# Resting state network changes induced by experimental inaudible infrasound exposure and associations with self-reported noise sensitivity and annoyance

**DOI:** 10.1038/s41598-024-76543-2

**Published:** 2024-10-19

**Authors:** Caroline Garcia Forlim, Leonie Ascone, Christian Koch, Simone Kühn

**Affiliations:** 1 Neuronal Plasticity Working Group, Department of Psychiatry and Psychotherapy, University Medical Center Hamburg-Eppendorf, Martinistraße 52, 20246 Hamburg, Germany; 2 Center for Environmental Neuroscience, Max Planck Institute for Human Development, Lentzeallee 94, 14195 Berlin, Germany; 3 Physikalisch-Technische Bundesanstalt Braunschweig, Bundesallee 100, 38116 Braunschweig, Germany

**Keywords:** Auditory system, Sensorimotor processing

## Abstract

The effects of prolonged infrasound (IS) exposure on brain function and behavior are largely unknown, with only one prior study investigating functional connectivity (FC) changes. In a long-term randomized-controlled trial, 38 participants were exposed to inaudible airborne IS (6 Hz, 80–90 dB) or sham devices for four weeks (8 h/night). We assessed FC changes in resting-state networks (auditory, default mode (DMN), sensorimotor (SMN), and executive control (ECN)), and explored IS ‘sensitivity’ as a predictor of identified significant FC changes. We also examined correlations between somatic symptoms and FC. IS exposure led to decreased FC in the right precuneus (DMN) and increased FC in the Vermis IV and V (SMN). In the ECN, we observed increased FC in the right frontal middle gyrus (BA8) and the right inferior parietal lobe, and decreased FC in another region of the right frontal middle gyrus. Changes in the ECN (right inferior parietal lobe) were negatively associated with self-reported annoyance from IS/low-frequency noise. A significant negative association was found between FC changes in the DMN (right precuneus) and somatic symptoms. Our study is the first to investigate prolonged IS exposure effects on brain FC, revealing changes in the vDMN, SMN, and ECN, but not in the auditory network. Future studies should assess annoyance and sensitivity markers, fine-grained measures of somatic symptoms, and stratify samples by sensitivity to uncover individual differences in response to IS.

**Supplementary Information:**

The online version contains supplementary material available at 10.1038/s41598-024-76543-2.

## Introduction

Humans are regularly exposed to both natural and human-made infrasound (IS). Generally, IS is defined as sound below the frequency of 20 Hz, which roughly constitutes the lower hearing threshold of humans, although sound can be perceived even below this frequency. In our everyday environment, IS occurs mostly intermixed with noise in the low-frequency range (LFN), which is defined as (audible) noise with predominant frequencies in the range of about 10 Hz to 200 Hz^1^. Natural IS sources include, e.g., wind, thunderstorms, ocean waves, or earthquakes, while human-made sources include larger structures or industrial objects such as heat pumps, ventilation systems, wind farms, large motors, or heavy machinery. There is a growing popular interest in the effects of IS on humans due to the increasing occurrence of windfarms to produce renewable energy.

Infrasonic frequencies can be consciously perceived given high enough sound pressure levels (SPLs), however the percept is not a ‘classical’ auditory perception, since tonality is lost. IS is reported to be perceived via body resonance phenomena^2^ or eardrum pressure^3^. Interestingly, acoustic experiments suggest that auditory perception in the infrasonic range shows high interindividual variability^4,5^. More specifically, individuals are differentially sensitive to hearing and judging IS, whereby hearing thresholds for the same frequency can differ by up to 20 dB. In addition, the subjective perception of loudness as well as annoyance ratings for the same IS stimulus can largely differ. Thereby, for instance, the same sound might not even be heard by one participant, while another may find it extremely loud and annoying^4^. It is hence well possible that a generalized sampling approach and averaging responses across participants might not aid in detecting (adverse) IS effects.

It has been suggested that such responses may apply to a small albeit significant minority of the population. For example, it has been reported that about 2.5% of middle-aged individuals, for yet unknown reasons, have a heightened sensitivity to IS and LFN, including a lower detection threshold (by SPL) and annoyance-responses to the same stimulation^1^. A review reported that the pooled prevalence of high annoyance by LFN, including IS, in everyday life in individuals living in the vicinity of according sound sources (e.g., wind farms) was about 10%^6^.

Concerning the effects of IS on humans, subjective and objective measures can and should be differentiated. Most of the studies into this direction are observational studies on windfarms. Thereby, it has been shown for example that IS/ LFN emissions are related to self-reported annoyance^7,8^ and, even in a dose-response manner, to self-reported stress and sleeping problems^9–11^. Critically however, the effect of IS exposure per se in these studies remains unstudied. Additionally, according to previous research a multitude of confounding factors need to be taken into account as well, such as visual impacts of the windfarms (shadows, blinking light), political attitudes, or (lack of) adequate or biased information about infrasound, and participation in decision-making processes^12–14^, or even nocebo effects^15,16^.

Studies specifically addressing causal objective, physiological effects of IS on humans are scarce. Generally, IS, albeit not unequivocally, has been linked at SPLs above 75 dB to symptoms such as cardiovascular or concentration problems, as well as vestibular symptoms^17^. In a particularly well-designed, controlled laboratory study, participants were seated in a living-room setting within an isolated facility and exposed to 30 min of audible or ambiguous (close to the hearing-threshold) infra- or LFN (3 Hz–1 Hz amplitude modulated, or 5 Hz – 105 dB, or 10 Hz – 95 dB, or 18 Hz – 85 dB). No acute physiological responses (blood pressure, heart rate, brain electrical activity in electroencephalography (EEG); neurological tests assessing e.g. vestibular responses/ balance, nystagmus) were observed. All IS stimuli, if audible to the subject, were judged as annoying and unpleasant by the participants. Thereby, annoyance was not correlated with previous IS exposure in daily life (speaking against sensitization or habituation). Inaudible IS was not perceived as annoying or unpleasant^17^.

Another important aspect of how IS affects humans is its influence on our brain, since this might constitute the underlying pathway by which annoyance may be generated. Thus, in the following, studies using brain imaging approaches are summarized with an emphasis on studies where functional magnetic resonance imaging (fMRI) has been used. MRI technology has several advantages, as it is non-radioactive, noninvasive and has a very high spatial resolution, including both cortical and (beyond the possibilities of EEG) subcortical areas that can reliably be measured. This enables the assessment of whole brain functional changes, i.e., brain activity and network reconfiguration. A first fMRI study in the field conducted by Dommes and colleagues^18^, revealed bilateral auditory cortex (AC) (BA 41, 42, and 22) activation by (audible) 12 Hz stimuli (110 and 120 dB) in *N* = 17 volunteers. This could not be replicated for 12 Hz inaudible IS (at 90 dB), except in one subject. Another study, the only one to explore functional connectivity, showed that brain areas involved in stress processing (right amygdala within the sensorimotor network (SEN)) exhibited changes in connectivity during a 12 Hz near-threshold (-2 dB below the individual hearing threshold) vs. both, a supra- threshold stimulus (medium subjective loudness of 122 dB) and a ‘no tone’ control condition in a sample of *N* = 14 volunteers^19^. In the same sample, enhanced bilateral AC activation (Brodmann area 41, 42) while performing a working memory task under (audible) short sinusoidal tone burst exposure of 12 Hz vs. no sound was observed, and there were trend-level improvements in task performance under IS^20^. In yet another study, IS and LFS were applied (8 Hz vs. 32 Hz, respectively) with varying SPLs, such as to examine whether there was an association between physical (frequency, SPL) stimulus properties and AC activation. The study revealed that both IS and LFN elicited similar AC activations, however whereby individual loudness perceptions, not physical stimulus properties, were related to the strength of the activation at an intraindividual level^21^. This finding suggests that individual characteristics, rather than physical sound properties, may amplify neuronal responses to IS.

In sum, existing fMRI research has shown that short-term IS exposure with sound pressure levels (SPLs) above the hearing threshold alters brain activity and connectivity in the AC, whereby there was no clear evidence for inaudible IS. Possibly, ‘sensitive’ individuals may respond more strongly to subthreshold exposure to IS. There were also indications that inaudible IS may influence the connectivity in affect- and attention-related networks in a single prior study. Against this background, we argue that (a) long-term exposure designs involving inaudible IS, and (b) data-driven approaches to detect brain connectivity changes including bot auditory and non-auditory networks could provide valuable insights in the field. In addition, since strong interindividual variability has been observed in response to IS, measures to capture ‘sensitivity’ to IS may be of particular interest as a potential predictor of change.

To the best of our knowledge, the first study in which participants were experimentally and longitudinally exposed to inaudible airborne IS (6 Hz, 80–90 dB) for four weeks with 8 h/ night (vs. sham), (full trial protocol available here: https://clinicaltrials.gov/study/NCT03459183, trial identifier: NCT03459183, ID: SonicBrain01) was conducted by the authors (LA, SK, CK). In the first publication on data of this trial^22^ based on exploratory analyses changes in brain anatomy (grey matter volume) following long-term IS exposure were investigated. Exploratory results showed decreases in regional grey matter volume (rGMV) in bilateral cerebellum (region VIIIa) and left angular gyrus for the IS group compared to sham. Additionally, there was also a trend-level increase in self-reported somatic symptoms, although those were unrelated to the observed rGMV changes. Importantly, no further effects due to long-term IS were identified across a large number of cognitive and self-reported (mental) health outcomes that were assessed, suggesting overall an absence or rather subtle overall effects of IS exposure^22^.

In the present study, we set out to investigate possible changes in brain function due to long-term exposure of inaudible IS using resting state fMRI data from the same long-term interventional randomized-controlled trial mentioned above. More specifically, we looked into possible whole brain network reconfigurations of functional connectivity (FC) using independent component analysis (ICA)^23^. The primary advantage of Independent Component Analysis (ICA) over other popular methods, such as seed-based connectivity, is its data-driven nature. ICA does not require prior assumptions about seed regions or rely on brain parcellations. This feature is particularly relevant for the current exploratory analysis, given the limited literature on the long-term effects of infrasound (IS) on brain function. Without a specific hypothesis regarding changes in functional connectivity due to infrasound, ICA emerges as the ideal methodological choice, enabling an exploratory, data-driven investigation.

We were particularly interested in changes within the following resting-state networks (RSN): auditory, default mode (DMN), sensorimotor (SMN) and executive control network (ECN). Besides the obvious choice of the auditory network, we selected the DMN, since it has been implicated in many studies on psychiatric disease and mental health issues. It comprises the dorsal medial prefrontal cortex, posterior cingulate cortex, precuneus, and angular gyrus. The DMN is a very ‘basic’ RSN, because it is the main network active during rest. SMN and ECN in contrast are known to respond to external inputs and therefore could potentially also be affected by IS. Namely, SMN comprises the pre- (motor cortex) and postcentral (somatosensory) gyrus, as well as auditory areas (superior temporal lobe) and the cerebellum, and is thus central to body sensation and regulation, perception, and motor action. The ECN on the other hand comprises fronto-parietal brain areas. Its intrinsic activity is related to problem-solving, conscious manipulation of information, or decision-making in working memory, and it is generally involved in mostly externally-driven functions. For a review please refer to^24^. In addition, regions within the DMN, SMN and ECN had shown changes in FC due to short-term IS exposure in the only previous study on IS exposure on functional connectivity in resting state networks^19^.

Motivated by the suggestions provided by Behler and colleagues^21^ that individual characteristics, rather than physical sound properties, may amplify neuronal IS responses, and to shed further light on the potential modulating effects of ‘sensitivity’, any identified significant changes in FC were correlated with baseline self-reported IS sensitivity, self-reported annoyance by existing IS sources in the environment, general audio sound/ noise sensitivity, and somatization since somatization showed trend-level changes after IS exposure^22^ .

## Methods

### In- and exclusion criteria and study design

The trial was pre-registered in the National Institute of Health trial registry: (full trial protocol available here: https://clinicaltrials.gov/ct2/show/NCT0345918; trial identifier: NCT03459183 (ID: SonicBrain01; registration date: 08/03/2018; recruitment and data collection took place between May 31, 2018 and December 15, 2019). In all procedures, we adhered to the declaration of Helsinki and the study was approved by a local ethics board prior to study onset (Ethik-Kommission der Ärztekammer Hamburg; approval number: PV5570). Informed consent was obtained from all study participants who were enrolled in the study before testing. The study was a 2 (infrasound verum vs. sham) × 2 (pre-post one month of sound exposure) repeated-measures randomized-controlled, single-blind experiments, with participants being unaware of group assignment, which was realized via list-wise randomization to either a sham or verum IS source. Included participants were sequentially assigned to the next available list position based on the list. The experimenter was aware of the group assignment.

An online screening was conducted prior to inclusion (duration ca. 45 min), which assessed socio-demographic data, including sex, age (required to be between 18 and 45 years), education, partnership status, children, regular medication intake, and variables addressing housing, as well as sleeping-room conditions (for later targeted source installation). Children were not allowed to sleep in the same bedroom as participants for safety reasons, and pet owners were advised to keep their animals outside the room for the time of the exposure. Main exclusion criteria were counter-indications for magnetic resonance imaging (MRI) (i.e., cochlear implants, non-removable metal on/ in the body, or tinnitus), chronic inflammatory, autoimmune, or other severe illnesses (e.g., cancer), as well as central-nervous-system diseases. Self-reported anomalies concerning hearing (e.g., deafness, past ear surgery, chronic inflammation of the ear canal, chronic sinusitis, anatomic anomalies), medication intake affecting the central nervous system, or participation in a medical trial led to exclusion. Assessed health-relevant variables were smoking and alcohol consumption. Mental illness was excluded by screening participants for past or present symptoms. Any positive answer indicating mental illness was followed up on in a subsequent telephone interview. Suspicion of a potential mental disorder led to exclusion from the study^22^.

**Fig. 1:**
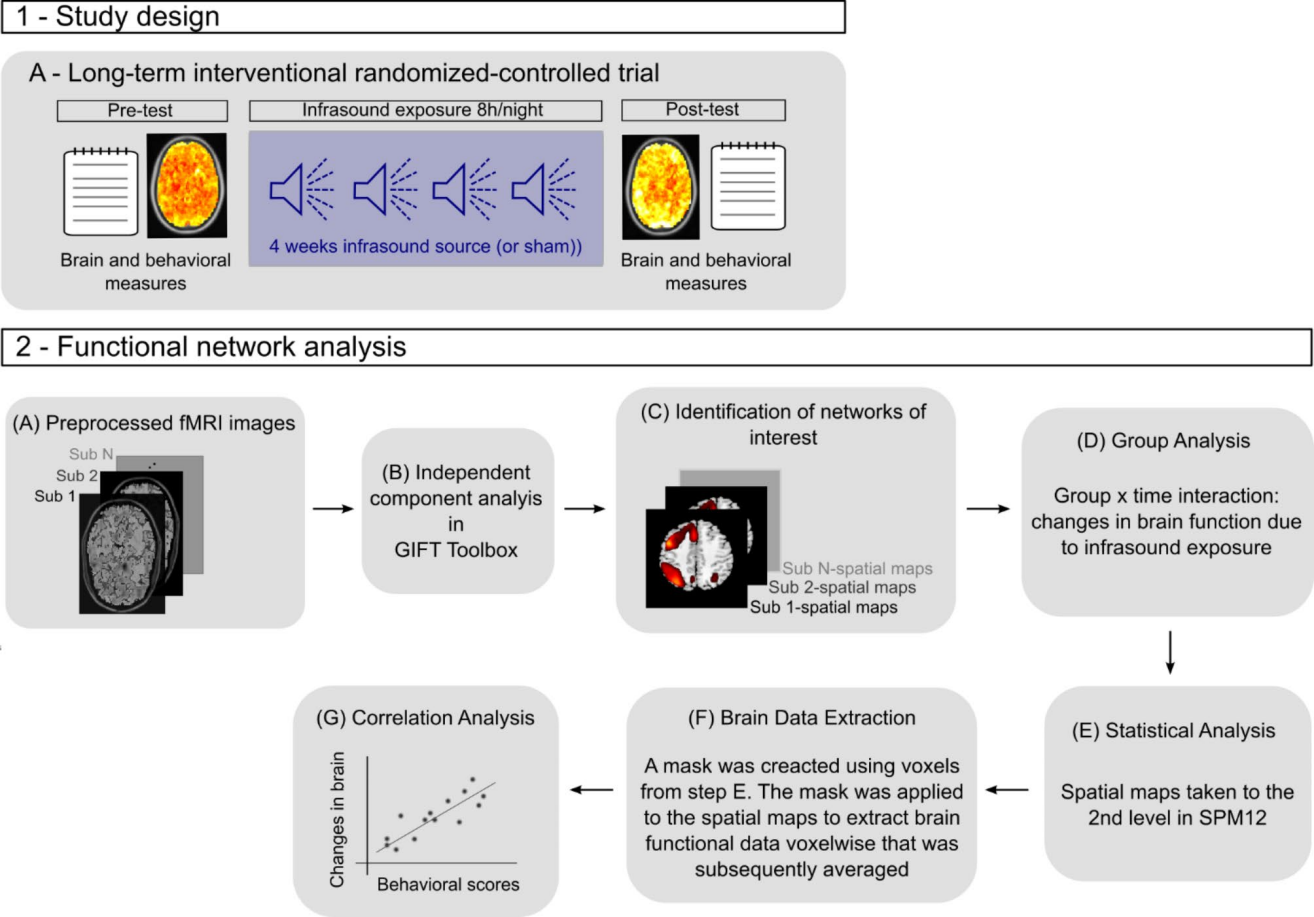
Part 1 - Study design: participants were exposed to infrasound (IS) between 80 dB and 90 dB (6 Hz frequency) for eight hours during their self-reported habitual sleep (bed) time interval. Brain and behavioral measures were collected before (pre-test) and after (post-test) IS exposure. Part 2 - Analysis workflow.

If all in- and exclusion criteria were fulfilled, appointments for the pre-test, on-site sound source installation, and post-test were made. Closely after the pre-test assessment (1–3 days to maximally one week after initial assessment), the on-site sound source installation took place, following a strict protocol for calibration and instruction of participants (for details see^22^, for technical details see the appendix of the first publication on this trial: https://static-content.springer.com/esm/art%3A10.1038%2Fs41598-021-82203-6/MediaObjects/41598_2021_82203_MOESM1_ESM.pdf). The IS emitted a steady SPL between 80 dB and 90 dB (6 Hz frequency) for all participants (i.e., SPLs were not individualized) for eight hours during the participant’s self-reported habitual sleep (bed) time interval (Fig. 1). This range of SPL was chosen as it can be expected to be below the hearing threshold, which was tested in different laboratory settings (see appendix cited above) and confirmed on-site by the experimenter who also tested out different room constellations to make sure that the source remained inaudible under different conditions, necessary for a blinded study. Eighty dB to 90 dB SPL can be considered as a high acoustic load, hence enhancing the likelihood of finding potential effects. Furthermore, the SPL range chosen is about 25 dB higher than common IS emissions from wind parks (see for example^3,25^). The choice of 6 Hz was a compromise between sound source manufacturing possibilities and constraints, and the wish to have a signal with low frequency. The sham sources had an identical design, including a ventilator for cooling and LED-lights to indicate operating status. Before and after the exposure, assessments took place at our research laboratory unit at University Medical Center Hamburg-Eppendorf, whereby questionnaire assessments, intermixed with computerized cognition tests, were conducted. At each assessment point an MRI session (total duration between 1 and 1.5 h) took place. As part of the MRI session, individuals also performed a spatial n-back fMRI task.

The aimed sample size was 40, determined taking into consideration the previous experience of the principal investigator SK in neuroplasticity induced by environmental changes in comparable samples and a balance of available time and resources.

### Noise sensitivity self-reports

Normal sound hearing sensitivity was assessed using the Noise Sensitivity Questionnaire (noiseq) which has been reported to have excellent reliability^26^. The questionnaire comprises 35 statements (e.g., ‘*I can only focus on novel tasks in a quiet environment*’), rated on 4-point Likert scales (strongly agree = 3, slightly agree = 2, slightly disagree = 1, and strongly disagree = 0). The total score across all items was used in the present study to reflect general (audio) noise sensitivity. Cronbach’s α in the present sample (total *N* = 38) was excellent (= 0.92).

Sensitivity to IS and LFN was assessed using the SISUS-Q (sensitivity to infra- and ultrasound questionnaire^27^) infrasound subscale, which is a brief and economic consisting only of four items (e.g., ‘*I can hear or otherwise perceive*,* e.g. via bodily sensations*,* low-pitched noises/sounds more often than other people/ I perceive them more intensely than others’*), rated on 11-point Likert scales (0 = *totally disagree*, 10 = *totally agree*). Cronbach’s α was poor in the present study sample (0.57), suggesting that the items were not strongly intercorrelated.

Furthermore, within the scope of the study annoyance by ten different IS/LFN sound sources in the environment (impact sound/ thuds; air traffic; subwoofer/ low-pitched instruments/ base drum; road traffic; industrial noise; washing machine; wind turbines; railway traffic; large ventilation systems; thunder) was assessed in a standardized manner. Annoyance was rated in line with ICBEN (The International Commission on the Biological Effects of Noise) guidelines^28^ (rating format from 0 to 10 [anchors: 0 = *does not bother/ annoy me at all*; 10 = *extremely bothers/ annoys me*) for each of the ten IS sources. The IS/LFN sound aspect/quality that needed to be rated was explicitly referred to for each sound source (e.g., ‘deep rumbling’, ‘vibrations’, ‘deep humming’, ‘deep swooshing’ etc.), for the participant to focus on the LFN/IS-related sound properties. ‘Not applicable’ could be indicated if the respective sound was never heard/ perceived and thus could not be evaluated. The mean score across the annoyance ratings served as a proxy for annoyance by environmental IS sources. Cronbach’s alpha for this ‘annoyance by environmental LFN/IS sources’ scale was excellent (= .92).

### Somatic responses

The Brief Symptom Inventory (BSI) somatization subscale was used to correlate any identified resting state network changes with changes in somatization (e.g., dizziness, bodily weakness, nausea), since a trend-level for a group by time interaction effect had been identified in a previous publication on this dataset showing an increase of the somatization subscale in the IS group^22^. This subscale contains 7 symptoms, which are rated on a 5-point Likert scale (*0 = not at all*, *4 = extremely*). Participants were instructed to rate the presence of such symptoms (vertigo/ fainting, heart/ chest pain, nausea/ stomach problems, respiratory problems, heat/ chills, numbness/ tingling sensation in parts of the body, weakness in certain body parts) for the past two weeks at baseline and at post-test, after the 1-month-verum vs. sham IS exposure. Cronbach’s alpha for this subscale was poor (0.54) in the present sample, suggesting that the items were not strongly intercorrelated.

### MRI data acquisition

Brain scans were performed on a 3T Siemens Magnetom Prisma (Siemens Medical Systems, Erlangen, Germany) using a 64-channel head coil. A sagittal MPRAGE (256 slices per slab, FOV = 240 mm, TR = 2500 ms, TE = 2.12 ms, TI = 1100ms, voxel size = 0.8 mm x 0.8 mm x 0.9 mm) was administered. A T2-weighted, BOLD sensitive resting state EPI sequence was run, with participants lying relaxed and awake (eyes open) in the scanner (TR = 2000 ms, TE = 30 ms, image matrix = 64 × 64, FOV = 216 mm, flip angle = 80º, voxel size 3 × 3 × 3 mm3, 36 axial slices).

### Functional MRI data analysis

#### Preprocessing

To ensure steady-state longitudinal magnetization, the first 10 images were discarded. We followed the standard preprocessing steps for rs-fMRI with related default settings in the SPM12 toolbox. First, the data was corrected for timing error using the slice timing option and then it was corrected for misalignments using ‘realign’. The next steps align both functional and structural images: the segmentation structural images into different classes (gray matter, white matter, cerebrospinal fluid), and moving them into a standardized space so that group analysis can be performed. Data was spatially normalized to the MNI template. Finally, the images were spatially smoothed with a 6-mm FWHM to improve signal-to-noise ratio. In addition, to control for motion, we used the voxel-specific mean frame-wise displacement (FD)^29^. All subjects had FD values lower than the default threshold of 0.5 mm. All steps were conducted using MATLAB 2016b (www.mathworks.com).

#### Network analysis: RSN from independent component analysis (ICA)

To extract the resting-state networks, ICA analysis was performed in the Group ICA Of fMRI Toolbox (GIFT) toolbox (http://icatb.sourceforge.net/)^23^. ICA is a data-driven technique that blindly recovers source signals from a mixture of sources. When applied to resting-state fMRI (rs-fMRI), ICA blindly decomposes the brain activity into multiple independent components. The independent components can either be related to sources of coordinated brain activity by spatial grouping of voxels with temporally coherent activity (= connectivity) or to random noise, such as movement, blinking, breathing, and heartbeat, depending). The spatial grouping of voxels with temporally coherent activity is referred to as spatial maps. The independent components related to sources of brain activity resemble discrete cortical functional networks, termed RSN. The RSN include the default mode network (DMN), auditory, language, visual, visuospatial, basal ganglia, salience, SMN, and ECN networks.

In the GIFT toolbox, we ran ICA using the Infomax algorithm. First, the optimal number of spatially independent resting-state networks (N) were estimated as 29. Then, ICA was run 29 times and the results were clustered by the GIFT toolbox ICASSO (http://research.ics.aalto.fi/ica/icasso/), with a minimum cluster size of 23 and a maximum of 29 (number of runs), whereby RandInit and Bootstrap were selected. Only components with an ICASO stability index Iq of > 0.9 were considered. The identification of the spatial maps of the RSN was done first automatically using predefined GIFT templates and a-posteriori by two specialists (CGF and SK). The RSN of interest identified were the auditory network, dorsal and ventral DMN (dDMN and vDMN), SMN and ECN. Their accoring subject-specific spatial maps were taken to the second level analysis in SPM12 using a mask for individual networks (provided by http://findlab.stanford.edu/functional_ROIs.html).

#### Brain-behavior correlations

If a significant change in verum vs. placebo (dummy exposure) was identified in any region within any of the RSNs of interest, the magnitude of the FC was extracted from the respective region in the spatial maps. In more detail, once these regions were identified, we used SPM to create a mask. This mask works by indicating the position of the voxels that correspond to the brain region of interest (i.e., in which the changes in FC occurred in verum vs. sham). Finally, we read in the position of the voxels in the brain via the mask and extracted the ICA values of the indicated voxel positions in the spatial map of the networks. More specifically, for each subject we extracted the absolute mean ICA value (which indicates the magnitude (or strength) of RSN connectivity), once for baseline, once for post-test. A difference score (post-test minus baseline) absolute mean ICA value was computed to create a single change score value of connectivity for each participant. Since the results from the previous step (network analysis RSNs using ICA) would establish differential change in verum (relative to no change in placebo), subsequent correlation analyses were limited to the verum group.

Thereby, first correlations between baseline noise (audio, LFN/IS) sensitivity and changes in FC were conducted. This was done in the hope of being able to establish whether lower/ higher sensitivity would relate to differential directions (connectivity in- vs. decrease) or magnitudes (strength) of change in connectivity in how individuals exposed to IS responded to it. Identifying such an association could indicate modulation of change by sensitivity and potentially aid in the selection process of participants in future trials. Additionally, change-to-change correlations between changes in connectivity and somatization were conducted to see whether there were any systematic patterns of corresponding change.

### Statistical analyses

#### Network analysis: RSNs from independent component analysis (ICA)

Subject-specific spatial maps were taken to the second level analysis in SPM12 using a flexible factorial design with a ‘subject’ factor as main effect, and including the group x time interaction, using a mask for individual networks (provided by http://findlab.stanford.edu/functional_ROIs.html). Movement inside the scanner given by the mean frame-wise displacement^29^ was used as covariate. The resulting maps were thresholded with *p* < .001 uncorrected, with an additional threshold of *p* ≤ .05 family-wise error rate (FWE) corrected on the peak level and an extent threshold of *≥* 15 voxels. We investigated group x time interactions in the RSN of interest, whereby we were interested in and modelled stability (no change) in the placebo condition and relative increases/decreases in the verum group.

#### Brain-behavior correlations

Correlation analysis was carried out using SPSS version 27 (IBM SPSS Statistics for Windows, Version 27.0. Armonk, NY: IBM Corp). If the Shapiro-Wilk test was non-significant (*p* > .05), Pearson correlation was applied, if *p* < .05, Spearman rank correlations were computed. Baseline sound sensitivity variables were associated with changes in connectivity in the identified clusters. In addition, changes in somatization were correlated with changes in identified clusters. Any significant associations were plotted using the GGRAPH scatterplot function and examined.

## Results

We first present the RSNs of interest, then we show regions within the RSNs which had altered function due to long-term IS exposure. Lastly, we show the association between network alterations and behavior (sound/ noise sensitivity and somatization). The analysis workflow can be seen in Fig. 1. Complete (after dropouts) pre- and post-test brain data was available for all participants in verum (*n* = 23; mean age = 27.4, SD = 6.44; 10 identifying as male and 13 as female) and placebo (*n* = 15; mean age = 25.6, SD = 4.76; 5 identifying as male and 10 as female). In total there were 5 dropouts: 4 in the pretest phase before group allocation and 1 after group assignment. There were no adverse events leading to premature study termination. Reasons for dropouts at pre-test were claustrophobia in the scanner (3 cases), and mental disorder (1 case) that was revealed during pre-test. The dropout after baseline (completed pre-test) was due to pregnancy (1 case—exclusion after sound source installation—placebo group). For full sociodemographic information and results concerning all behavioral outcomes as well as grey matter volume, please refer to Table 1 in Ascone et al. (2021)^22^. There were no significant differences in any of the demographic variables across the groups at baseline.

### Network analysis: RSNs from independent component analysis (ICA)

**Fig. 2:**
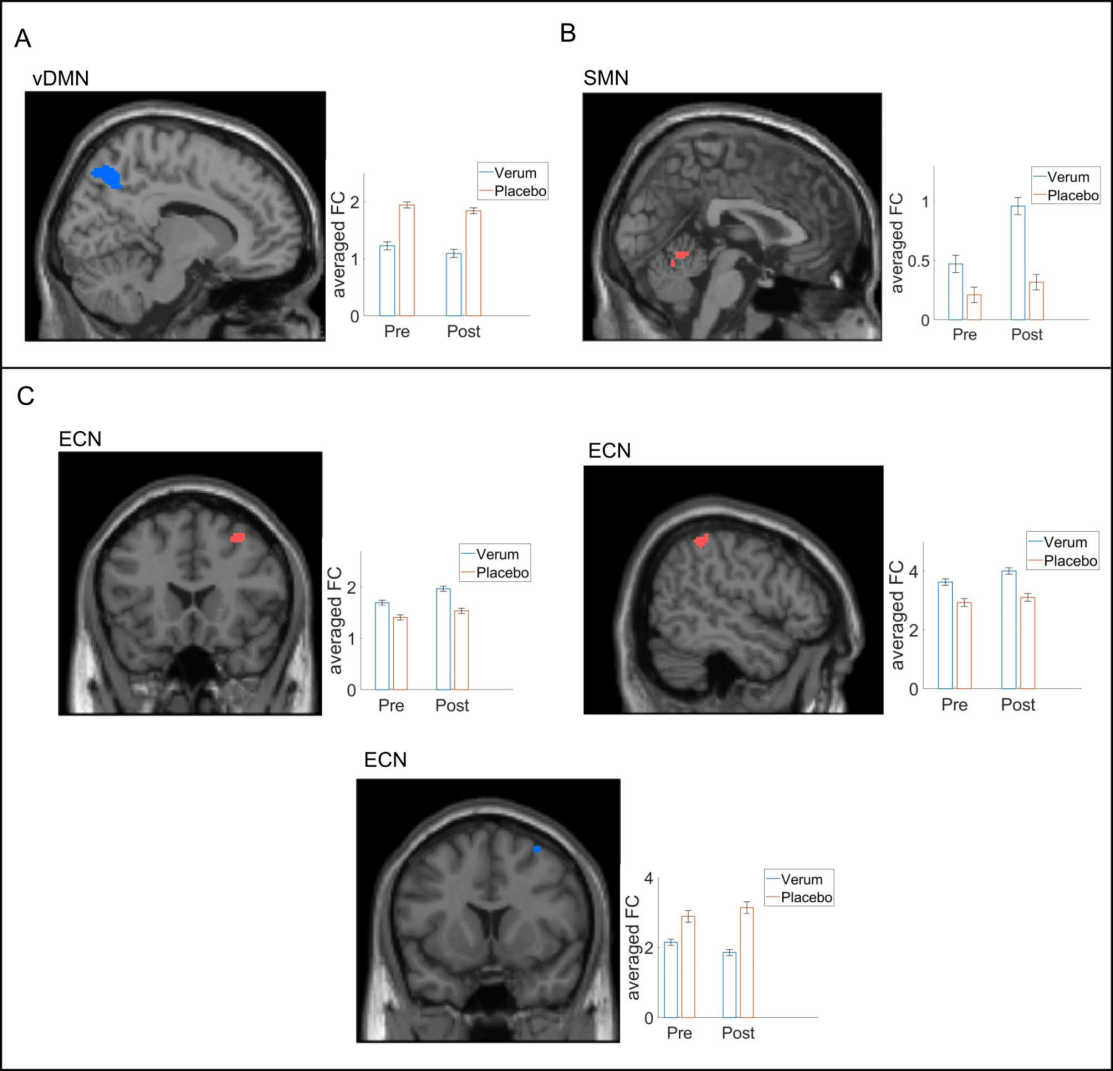
Functional connectivity (FC) changes (group x time interaction) in resting state networks (RSNs) due to long-term infrasound (IS) exposure (blue color corresponds to decreases in FC and red color to increases in FC. A-decreased FC in the right precuneus within the ventral default mode network (vDMN). B-increased FC in the Vermis IV and V within the sensorimotor network (SMN). C-changes in FC within the executive control network (ECN): increased FC in the right frontal middle gyrus (BA8) and right inferior parietal lobe (left and right middle panel); decreased FC in the right frontal middle gyrus (lower panel). No significant changes in FC were found within the auditory network and dDMN. Bar plots are presented for descriptive purposes only, showing the averaged FC changes. Please note that the statistical significance is calculated voxel-wise in SPM, and not on average, thus, averages do not necessarily represent the interaction effect.

For each individual the spatial maps of the RSNs of interest were calculated, i.e., for the auditory network, DMN (dDMN and vDMN), SMN and ECN. The mean spatial maps are shown in the supplementary materials (Figure S1). Group x time interaction effect analyses revealed functional changes in three networks: vDMN, SMN and ECN (see Fig. 2; Table 1). In the SMN, there was an increase in FC in lobule IV/ V of the cerebellar Vermis. Within the ECN, we observed both increased and decreased FC. Increased FC was seen in BA8/ right frontal middle gyrus and right inferior parietal lobe, whereas decreased FC was observed in the right frontal middle gyrus. In the vDMN, we found decreased FC in the right precuneus. No significant changes in FC were observed within the auditory network and dDMN.

**Table 1:** Group by time interaction within the RSN of interest. Abbreviations: ventral default mode network (vDMN), sensorimotor network (SMN) and executive control (ECN) network.

RSN	Brain region	MNI coordinates	T	Cluster size (in voxels)	*p*-value (FWE at peak level)	Direction of change in FC:
**vDMN**	Right Precuneus	12, −66, 50	6.65	41	<0.001*	Decrease
**SMN**	Vermis IV and V	2, −56, −8	4.77	21	0.049	Increase
**ECN**	BA8/ right frontal middle gyrus	32, 20, 50	5.09	40	0.016*	Increase
	right inferior parietal lobe	52, −44, 56	5.08	46	0.016*	Increase
	right frontal middle gyrus	34, 12, 62	5.29	15	0.010*	Decrease

^*^Survived multiple comparison correction (false discovery rate (FDR).

### Brain-behavioral correlation

**Fig. 3:**
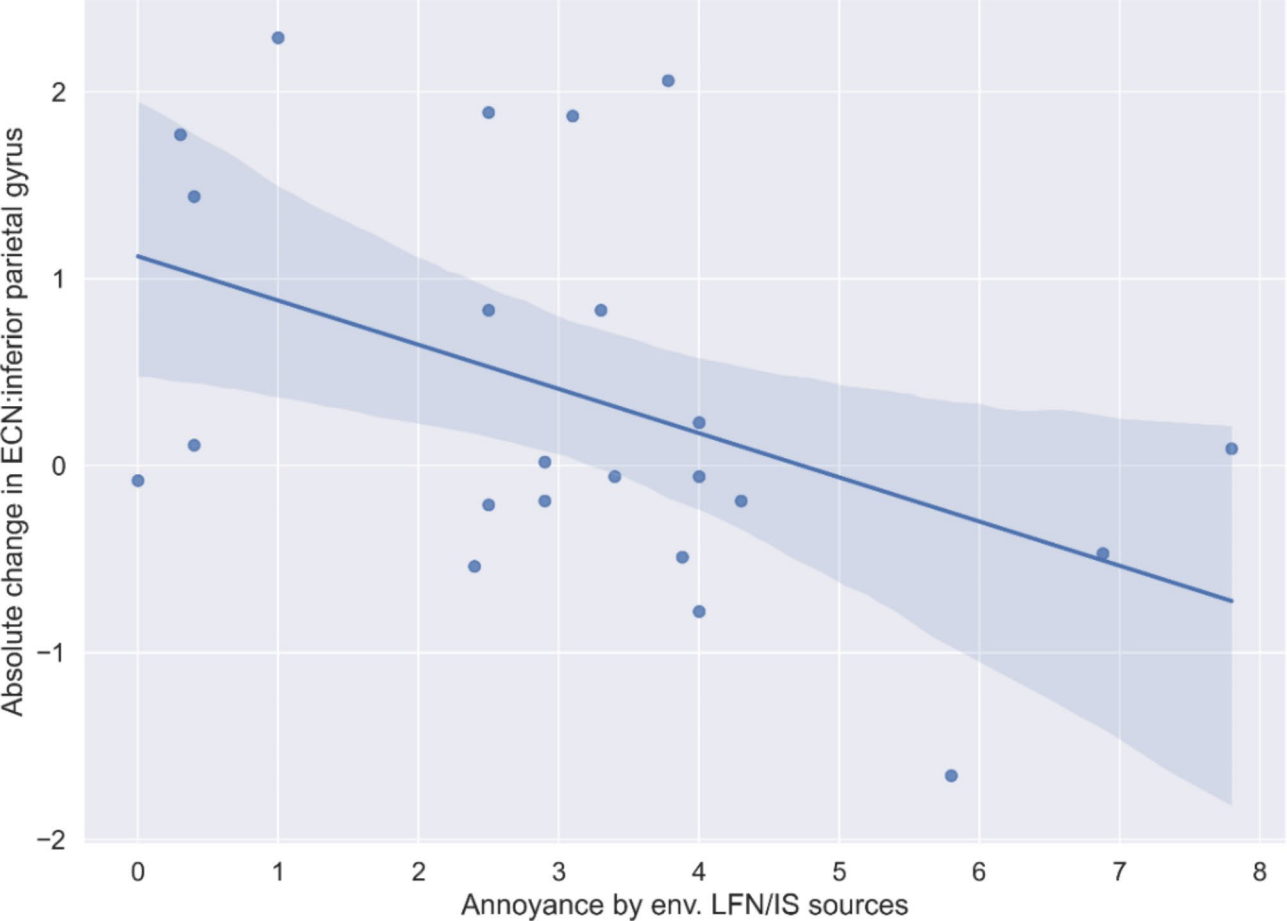
Depiction of the significant correlation between change in FC in a cluster within the executive control network and self-reported average annoyance by low-frequency/ infrasound sources in the environment within the infrasound verum group.

For all correlations and associated *p-values* please refer to Table 2. FC change in the ECN (right inferior parietal lobe, (52, -44, 56)) was negatively associated with average annoyance by different IS/LFN sources in the environment. The correlation is plotted in Fig. 3. As can be seen in the scatter plot, the more annoyed individuals were by environmental LFN/IS sound sources, the less they displayed an increase or even exhibited a decline in FC in the right inferior parietal lobe (ECN). There were no further significant associations between annoyance by LFN/IS or -sensitivity, general (audio) noise sensitivity and the observed changes in FC in verum.

**Table 2:** Correlation analyses to test for associations between baseline infrasound sensitivity and identified significant changes in resting state network connectivity within verum (*N* = 23). Abbreviations ventral default mode network (vDMN), sensorimotor network (SMN) and executive control (ECN) network.

Variable		Annoyance by env. IS/LFN sources	IS/LFN sensitivity (SISUS-Q)+	General noise sensitivity	Soma-tization
Right frontal middle gyrus/ BA8 within ECN	*ρ/r* *p*	− 0.203 0.353	0.000 1.000	− 0.140 0.523	− 0.362 0.90
Right inferior parietal lobe within ECN	*ρ/r* *p*	− 0.448* **0.042**	− 0.052 0.814	− 0.369^†^ 0.083	− 0.015 0.944
Right frontal middle gyrus 2 within ECN	*ρ/r* *p*	− 0.066 0.765	− 0.128 0.559	− 0.261 0.229	− 0.054 0.806
Vermis within SMN	*ρ/r* *p*	− 0.299 0.166	− 0.398† 0.060	− 0.332 0.122	0.054 0.806
BA7/ precuneus within vDMN	*ρ/r* *p*	− 0.305 0.157	0.119 0.587	0.276 0.203	− 0.423* **0.044**

*Note. Variable marked with+exhibited non-normality according to the Shapiro-Wilk-test (p < .05), in which case instead Pearson correlations (r), non-parametric Spearman-Rho (ρ) correlations were computed for any association with the variable; annoyance by environmental LFN (low frequency noise)/ IS (infrasound) sources = average annoyance by everyday-life low-frequency/ infrasonic sound sources; IS-LF sensitivity (SISUQ-Q) = self-reported general infrasound/ low-frequency sensitivity based on the Sensitivity to Infrasound and Ultrasound Questionnaire [22]; General noise sensitivity = sum score of noise sensitivity questionnaire (noiseq) by Schutte and colleagues [26], Somatization = subscale from the brief symptom inventory (BSI), for which a trend-level significant group (sham vs. verum exposure) x time (pre-post) interaction was found in the main publication of the experiment [22]. We report uncorrected p-values.*

**Fig. 4:**
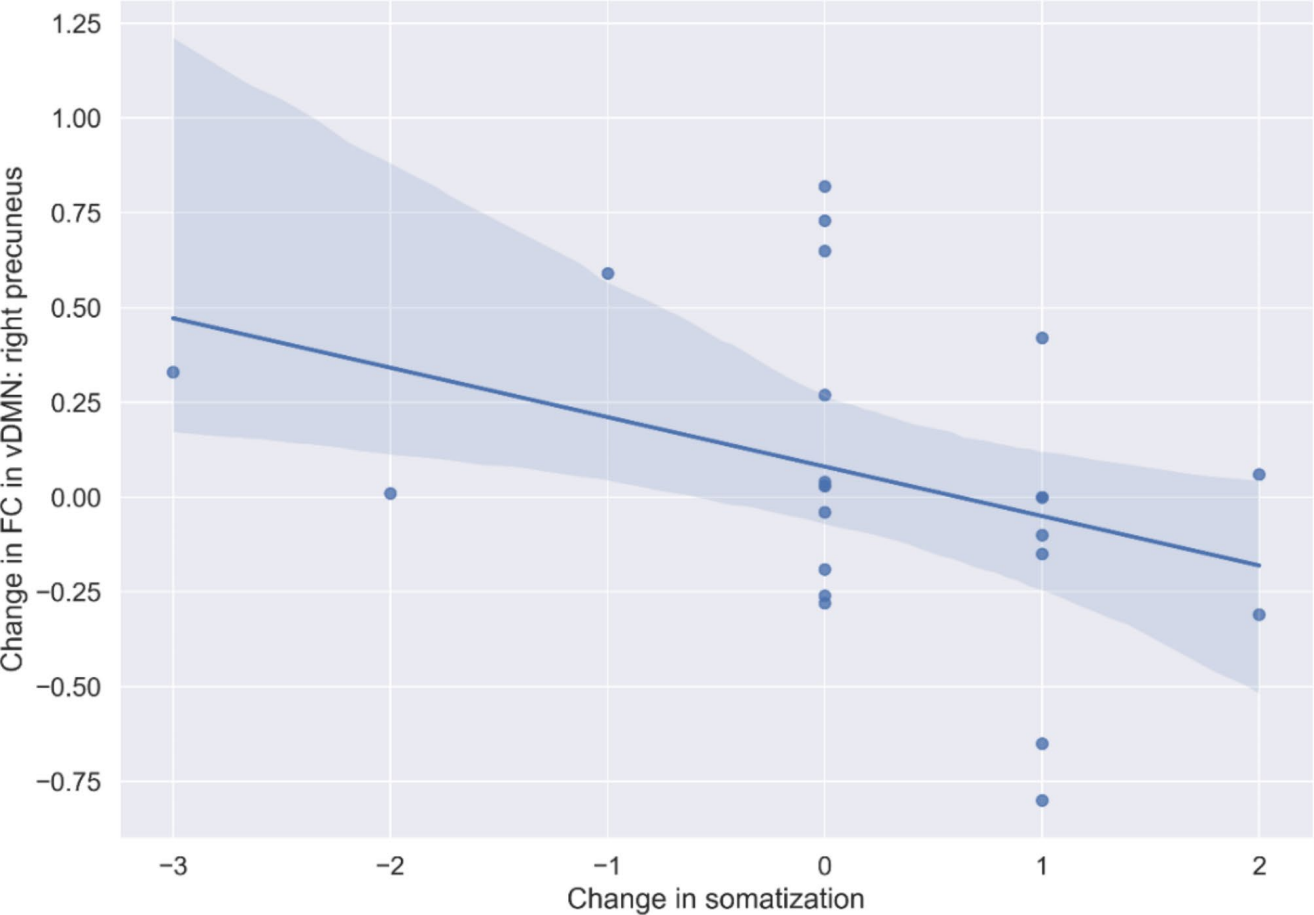
Depiction of the significant correlation between change in functional connectivity (FC) in a precuneus within the ventral default mode network (vDMN) and changes in self-reported somatic symptoms within the infrasound verum group.

Change in somatization (post-pre scores) were negatively associated with change in FC in right precuneus within the vDMN (12, -66, 50). Descriptive analyses of the scatter plots (Fig. 4), together with the underlying data revealed that individuals who had reported either a decrease (*n* = 3) or no change (*n* = 11) in somatization exhibited more increases in FC in the vDMN (*n* = 10 vs. *n* = 4), whereas individuals with an increase in somatization (*n* = 9) rather exhibited a decline in FC (*n* = 7 vs. *n* = 2) in the vDMN. Hence, possibly, the observed overall (relative to placebo) decrease in FC in this area characterizes individuals who, at the same time, tendentially exhibited an increase in somatic symptoms.

## Discussion

To our knowledge, the present study is the first randomized trial to investigate whether prolonged inaudible airborne IS exposure (6 Hz) affects FC at rest in the human brain. We found FC changes within three networks: vDMN, SMN and ECN. We showed decreased FC in the right precuneus within the vDMN, decreased FC in the Vermis IV and V within the SMN and within the ECN we observed increased and decreased FC. Both increased and decreased FC was observed in different parts of the right frontal middle gyrus (BA8) and increased FC was observed in the right inferior parietal lobe. No significant changes in FC were found within the auditory network and dDMN.

Despite the great public interest in possible effects of IS on the body and mind, the experimental neuroimaging literature, until the present day, is scarce. There are in total four fMRI studies on IS, three of which^18,20,21^ investigated brain activation with the attempt to understand where IS is processed in the brain. Only one study^19^ explored the effects of IS on functional connectivity. From brain activation studies it can be learned that IS activates similar brain areas as those activated for non-IS sound processing, mostly bilateral (primary and secondary) AC. Interestingly, Behler and colleagues (2009)^21^, who used an 8 Hz sinusoidal IS stimulus, reported that activation in AC was better predicted by individual perception than by the objective sound pressure level (SPL). Also worth of note is that the listeners’ judgment of loudness and unpleasantness was highly heterogeneous. Our results similarly show heterogeneity of ratings across participants for annoyance by LFN/IS sources (Fig. 3, ratings from 0 to 8 on a scale ranging from 0 to 11), which hints towards the importance of individual sound perceptions and appraisals in the prediction of both brain activation and functional connectivity changes.

Besides the current study, so far only Weichenberger and colleagues^19^ looked into functional changes in resting-state brain networks in response to IS exposure. Although the experimental paradigms are not directly comparable, it is noteworthy that we as well as Weichenberger et al. did not find changes in connectivity within the auditory network. However, we and Weichenberger et al. found differences in connectivity within the ECN^19^. From these results we conclude that the ECN network is affected by both short- and long-term exposure to IS. In sum, we can say that IS, as other sounds, may evoke activity in the AC during direct exposure^18,20,21^ but the functional involvement of the auditory network at rest is unchanged after both short- and long-term IS exposure^19^. However, functional changes do occur within the ECN, DMN and SMN. The ECN’s intrinsic activity is related to problem-solving, conscious manipulation of information, or decision-making – more generally-speaking brain function related to goal directed human behavior^30,31^. The SMN is central to body sensation and regulation, perception, and motor action^32^. The DMN is known to exhibit lower intrinsic activity upon task engagement, and higher activity when being awake but not engaged in any specific task (e.g., daydreaming)^30,33^. The precuneus is a central hub within the DMN and decreased FC in this region was observed in patients with schizophrenia and correlated with the negative psychotic symptom of apathy^34^. Nevertheless, the lack of significant behavioral changes reported in a prior publication on the present trial^22^ does preclude us from inferring that the decreased FC in the precuneus within the DMN is related to any negative health effects after long-term IS exposure. Moreover, taken together those findings contradict the assumption by Weichenberger and colleagues^19^ who suggested that, based on their identified FC changes in brain regions known to play a crucial role in stress (right amygdala, anterior cingulate cortex), IS exposure could potentially result in long-term symptom formation. Albeit no definite conclusions can be drawn concerning the behavioral implications of the identified resting state connectivity changes, the brain networks were reconfigured in circumscribed regions after IS exposure.

We identified two significant brain-behavior associations in the present study. First, the FC change in the right inferior parietal lobe within the ECN (general increases in verum vs. sham) was significantly (negatively) associated with self-reported infra- or low frequency noise sensitivity (higher rated annoyance by respective sound sources in the environment). Individuals with such higher self-reported annoyance by different IS/LFN sources in the environment showed less increases in FC or even a decline in response to IS in this area. Since the inferior parietal lobe in general is involved in attention and sensory integration, IS possibly changes these processes differentially in non-sensitive vs. sensitive individuals. Infrasound and low frequency noise sensitivity may hence be important factors to consider when conducting this type of experimental research. Albeit no negative effects of IS on (mental) health and cognition have been identified across a vast assessment battery in our previous study^22^, it is still possible that a subgroup of individuals may respond more strongly to IS exposure. Second, there was a significant negative association between changes in somatization and FC in the precuneus (decrease; within the vDMN) in the present study, which lead us to cautiously conclude that the observed overall relative decline in verum (vs. sham) in FC was strongest in individuals who also experienced an increase in somatization. Speculatively, this correlation could be interpreted from the background of the role of the precuneus in self-consciousness or self-related mental representations, including body awareness^35^, which in turn may relate to heightened sensitivity concerning physical symptoms in response to IS.

Mechanisms for how infrasound may affect the body, or more specifically the brain, have been proposed by different researchers, without reaching a definite conclusion. Persinger and colleagues^2^ for instance state that – given sufficiently high sound pressure levels – IS (in the frequency range (5–80 Hz) is directly absorbed by the body, stimulating body tissue, and often results in body vibration or resonance phenomena. These resonance phenomena also depend upon the respective organ dimensions and cavities, specifically resonating at certain frequencies. Moreover, sensory nerves and receptors, for instance somatosensory or visceral afferent nerve fibers may transmit signals to the brain, resulting in changes in connectivity. Mechanical stimulation by IS could also stimulate the displacement-sensitive cochlear outer hair cells (OHCs), which are sensitive to low frequencies outside the hearing range^36^. Signals from the OHCs are then transferred to the central nervous system via type II afferent fibers, first to the cochlear nucleus via several midbrain structures, to the thalamus and finally the auditory cortex, with further processing in higher-order cortical areas. As of now, we cannot say for sure which pathways may be responsible for the observed FC changes in the present study. Experimental research is necessary to further investigate potential translational mechanisms.

Mechanisms for how infrasound may affect the body, or more specifically the brain, have been proposed by different researchers, without reaching a definite conclusion. Leventhall^37^ discusses four different potential processes including the activation of the whole body which is also reported by persons in access to strong audio systems. It is, however, questionable whether the sensation stems from an impact of the sound wave or vibrations introduced directly by the sources or indirectly by an interaction of the sound wave with absorbing or reflecting objects within the sound propagation path. It cannot be excluded that such an interaction took place during our experimental setup and the change in SMN could be caused be a sensation of body-wide vibration. Further experiments should address this question by a direct application of a vibration during fMRI-measurements.

This study has several limitations that warrant caution in interpreting the results. The most significant limitation is the mixed pattern of individual responses combined with a small sample size, necessitating replication within larger samples. Another limitation involves the measure of somatic symptoms using a subscale of the Brief Symptom Inventory (BSI). The internal consistency of this scale was poor, and the 5-point Likert scale may have been inadequate for capturing more subtle changes, although it is also possible that indeed most individuals experienced no changes in somatic symptoms.

Future studies should employ more nuanced self-report assessments of physiological symptomatic somatic responses to infrasound (IS), such as by using visual analogue scale ratings (e.g., 0-100%). Additionally, a broader range of symptoms that are commonly reported by individuals affected by infrasound, such as dizziness, fatigue, sleep problems, reduced alertness, headaches, nausea, vertigo, chest resonance, palpitations, neck or back pain, shortness of breath, disorientation, pins-and-needles sensations, and ear pressure^1,38^, should be investigated thoroughly. These symptoms could also be additionally assessed directly after (nighttime) exposure to IS to assess short-term effects, as well as concerning persistent somatic or physical health effects. Incorporating objective physiological assessments, such as grip strength, electromyography, and (during sleep) actigraphy or EEG, would also have been beneficial alongside self-reports. Moreover, IS-sensitivity could have been assessed using audiometric testing, which would have been useful due to low reliability of the SISUS-Q IS-sensitivity subscale in the present study. These methodological improvements are important for future research. Lastly, we did not apply p-level correction in our correlational analyses to avoid overlooking potentially important associations, especially given the small sample size. This again underscores the need to interpret the findings of this study with utmost caution.

## Conclusion

Our study indicates that long-term exposure to inaudible infrasound (IS) at 6 Hz (80–90 dB) affects resting state network functional connectivity (FC) within the ventral default mode network (vDMN), sensorimotor network (SMN), and executive control network (ECN), which are not typically involved in emotional and autonomic control but relate to cognitive and sensory processing. This contrasts with the only previous study on infrasound (IS) exposure effects on FC, which found changes in the amygdala and anterior cingulate cortex during experimental exposure, suggesting a potential stress response. Notably, the auditory network was unaffected by long-term IS exposure in the present study. Significant changes were observed in the ECN, specifically an increase in FC in the right inferior parietal lobe during verum exposure compared to sham. This increase in FC was significantly and negatively correlated with self-reported annoyance from environmental low-frequency noise (LFN) or IS sources, implying that higher annoyance levels may attenuate the response to experimental IS exposure. Additionally, increased somatic symptom complaints following IS exposure were associated with stronger declines in FC in right precuneus, which overall showed a decline in FC during verum exposure relative to sham. Among other functions, the precuneus is involved in self-conscious mental processes, including body awareness, which makes the FC changes associated with somatization appear plausible, perhaps via changes in body perception. These findings highlight the need for further research on the effects of prolonged IS exposure and underscore the importance of assessing or stratifying participants based on different levels of self-reported annoyance or sensitivity to IS/LFN.

## Electronic supplementary material

Below is the link to the electronic supplementary material.

Supplementary Material 1

## Data Availability

For information about how to obtain the data please contact Prof. Dr. Simone Kühn.
