# Supplementary material for: Resting state network changes induced by experimental inaudible infrasound exposure and associations with self-reported noise sensitivity and annoyance
Source: Sci Rep. 2024 Oct 19;14:24555. doi: 10.1038/s41598-024-76543-2 (PMC11490626; doi:10.1038/s41598-024-76543-2)
Supplement: Supplementary file 1 — Supplementary Material 1 [file 41598_2024_76543_MOESM1_ESM.pdf]

Supplementary material:

Results

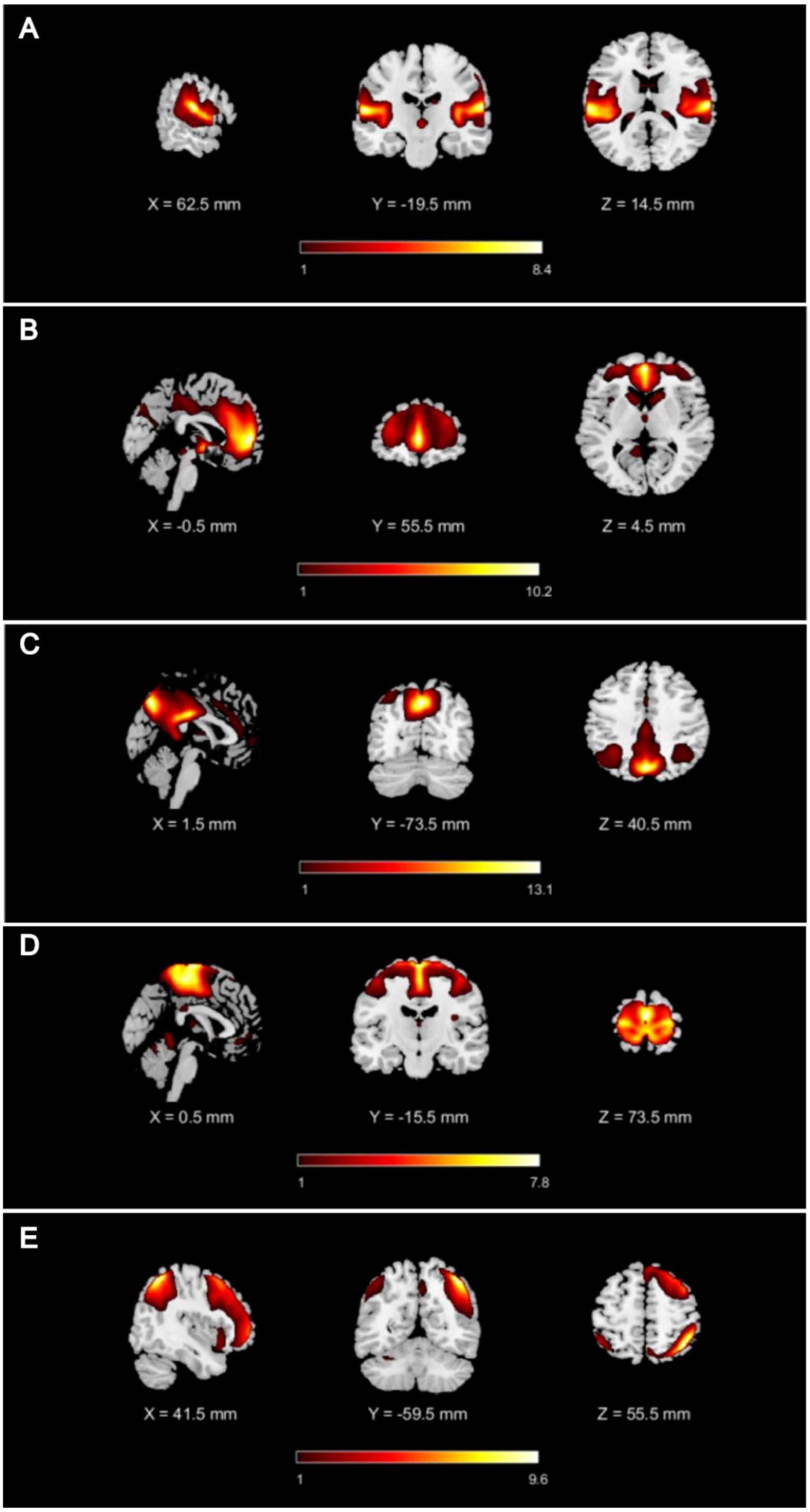

Figure S1 – Auditory network. B – Dorsal default mode network (dDMN). C – Ventral default mode network (vDMN). D – Sensorimotor network (SMN). E – Right executive control network (ECN). Colorbar represents z-score ICA values (functional connectivity).
